# Molecular Events Underlying Parkinson’s Disease – An Interwoven Tapestry

**DOI:** 10.3389/fneur.2013.00033

**Published:** 2013-04-08

**Authors:** Kah-Leong Lim, Cheng-Wu Zhang

**Affiliations:** ^1^National Neuroscience InstituteSingapore, Singapore; ^2^Duke-National University of Singapore Graduate Medical SchoolSingapore, Singapore; ^3^Department of Physiology, National University of SingaporeSingapore, Singapore

**Keywords:** Parkinson disease, mitophagy, autophagy, proteasome, oxidative stress, protein aggregation

## Abstract

Although a subject of intense research, the mechanisms underlying dopaminergic neurodegeneration in Parkinson’s disease (PD) remains poorly understood. However, a broad range of studies conducted over the past few decades, including epidemiological, genetic, and post-mortem analysis, as well as *in vitro* and *in vivo* modeling, have contributed significantly to our understanding of the pathogenesis of the disease. In particular, the recent identification and functional characterization of several genes, including α*-synuclein*, *parkin*, *DJ-1*, *PINK1*, and *LRRK2*, whose mutations are causative of rare familial forms of PD have provided tremendous insights into the molecular pathways underlying dopaminergic neurodegeneration. Collectively, these studies implicate aberrant mitochondrial and protein homeostasis as key contributors to the development of PD, with oxidative stress likely acting as an important nexus between the two pathogenic events. Aberrations in homeostatic processes leading to protein aggregation and mitochondrial dysfunction may arise intrinsically in substantia nigra pars compacta dopaminergic neurons as a result of impairments in the ubiquitin-proteasome system, failure in autophagy-mediated clearance, alterations of mitochondrial dynamics, redox imbalance, iron mishandling, dopamine dysregulation, or simply from the chronic pace-making activity of nigra-localized L-type calcium channels, or extrinsically from non-autonomous sources of stress. Given the myriad of culprits implicated, the pathogenesis of PD necessarily involves an intricate network of interwoven pathways rather than a linear sequence of events. Obviously, understanding how the various disease-associated pathways interact with and influence each other is of mechanistic and therapeutic importance. Here, we shall discuss some key PD-related pathways and how they are interwoven together into a tapestry of events.

## Introduction

Parkinson’s disease (PD) is a prevalent neurodegenerative movement disorder affecting 1–2% of the worldwide population above the age of 65 (Dorsey et al., 2007). Clinically, the disease is attended by a constellation of disabling motoric deficits including bradykinesia (slowness in movements), rigidity, and tremor that progressively worsen with age, ultimately leading to near total immobility. Although pathological changes are distributed in the PD brain (Braak et al., 2003), the principal lesion that underlies the characteristic motor phenotype of PD patients is unequivocally the loss of dopaminergic neurons in the substantia nigra pars compacta (SNpc) of the midbrain, which normally innervates the striatum. This specific pattern of neurodegeneration in PD is often accompanied by the presence of eosinophilic intracytoplasmic inclusions known as Lewy bodies (LBs) in surviving neurons in the SN and other affected brain areas (Braak et al., 2003). The depletion of striatal dopamine (DA) as a result of SNpc dopaminergic neuronal loss leads to an impaired nigro-striatal system that otherwise allows an individual to execute proper, coordinated movements. Accordingly, pharmacological replacement of brain DA via L-DOPA administration represents an effective symptomatic recourse for the patient (especially during the initial stages of the disease) and remains a clinical gold standard treatment for PD. However, neither L-DOPA nor any currently available therapies can slow or stop the insidious degenerative process in the PD brain. Thus, PD remains an incurable disease. Invariably, the debilitating nature and morbidity of the disease present significant healthcare, social, emotional, and economic problems. As the world population rapidly ages, these problems undoubtedly will also increase. This is definitely a worrying trend, and one that aptly emphasizes the urgency to develop more effective treatment modalities for the PD patient. Toward this endeavor, a better understanding of the molecular mechanism(s) that underlies the pathogenesis of PD would certainly be helpful, as the illumination of which would allow the identification and therapeutic exploitation of key molecules/events involved in the pathogenic process.

Although a subject of intense research, the mechanisms underlying PD pathogenesis remain incompletely understood. However, a broad range of studies conducted over the past few decades, including epidemiological, genetic, and post-mortem analysis, as well as *in vitro* and *in vivo* modeling, have contributed significantly to our understanding of the pathogenesis of the disease. In particular, the recent identification and functional characterization of several genes, including α*-synuclein*, *parkin*, *DJ-1*, *PINK1*, and *LRRK2*, whose mutations are causative of rare familial forms of PD have provided tremendous insights into the molecular pathways underlying dopaminergic neurodegeneration (Martin et al., 2011). Notably, the clinical manifestations and neuro-pathology of familial parkinsonism can often be quite indistinguishable from sporadic cases, which fueled the widely held assumption that the two forms of PD are likely to have shared pathogenic mechanisms. Collectively, these studies consistently implicate aberrant protein and mitochondrial homeostasis as key contributors to the development of PD, with oxidative stress likely acting as an important nexus between the two pathogenic events. Further, emerging evidence also implicates a host of new pathways, including impairments in vesicular dynamics, calcium homeostasis, and lipid metabolism that might contribute to disease pathogenesis in a manner that is not necessarily uncoupled from one another or from protein and mitochondrial homeostatic processes. Thus the molecular events underlying dopaminergic neurodegeneration in PD appears interwoven and complex. Here, we shall review some key PD-related events and discuss their reciprocal effects on each other.

## Aberrant Protein Homeostasis and PD

**Figure 1:**
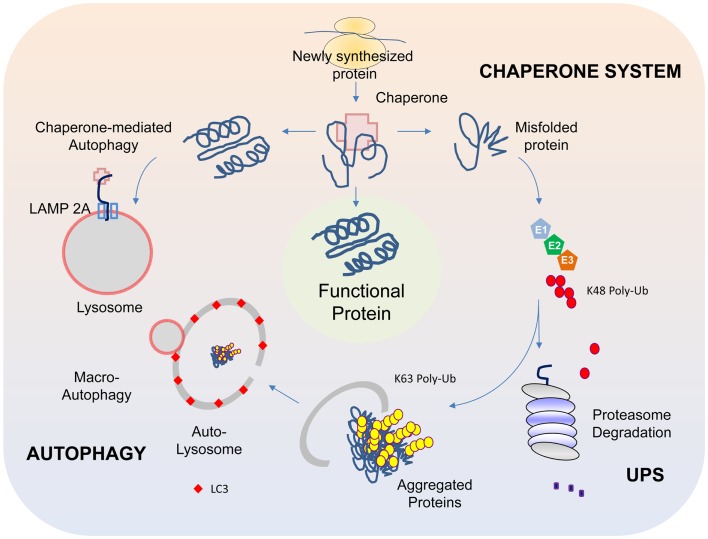
**Protein QC systems**. The chaperone, ubiquitin-proteasome, and autophagy systems function co-ordinately to maintain intracellular protein homeostasis. The chaperones, comprising of members of the heat-shock proteins, represent the first line of defense in ensuring the correct folding and refolding of proteins. When a native folding state cannot be attained, the chaperones will direct the misfolded protein for degradation by the proteasome. Proteins that are destined for proteasome-mediated degradation usually have a chain of ubiquitin added via a reaction cascade that involves the ubiquitin-activating (E1), -conjugating (E2), and -ligating (E3) enzymes, whereby successive iso-peptide linkages are formed between the terminal residue (G76) of one ubiquitin molecule and a lysine (K) residue (most commonly K48) within another. The (G76-K48) polyubiquitinated substrate is then recognized by the 26S proteasome as a target for degradation. In some cases, proteins may be modified by K63-linked polyubiquitination, which can promote their aggregation into inclusion bodies and their subsequent removal by autophagy. The autophagy process involves the sequestration of substrates by a phagophore that expands into a double-membrane structure called autophagosome that engulfs the substrate. The autophagosome then fuses with a lysosome to form autolysosomes, within which the inner membrane of the autophagosome is broken down and the cargo degraded by acidic lysosomal hydrolases. Another form of autophagy is chaperone-mediated autophagy (CMA), which involves the direct translocation of unfolded substrate proteins across the lysosomal membrane through the actions of a cytosolic chaperone hsc70, and an integral lysosomal membrane receptor LACMP2A (lysosome-associated membrane protein type 2A).

The presence of LBs in affected regions of the PD brain in numbers that far exceed their occasional presence in the normal brain arguably provides the most glaring evidence suggesting that protein homeostasis has gone awry during disease pathogenesis. As the cell is endowed with several complex surveillance machineries (including the chaperone, ubiquitin-proteasome, and autophagy systems) that rapidly detect and repair faulty proteins, and also destroy those that are beyond repair (Figure 1) (for a recent review, please refer to Tan et al., 2009), the presence of LBs suggests that these homeostatic response systems have failed in one way or another in the PD brain. Support for this came from various groups following the identification of α-synuclein, a presynaptic terminal-enriched protein that is prone to misfolding and aggregation, as a major component of LBs (Spillantini et al., 1997). Accordingly, elucidating how α-synuclein interacts with the various protein quality control (QC) systems to result in LB biogenesis would be an important cornerstone upon which a better understanding of PD pathogenesis could be built.

We now know that all the three protein QC systems (i.e., chaperone, UPS, and autophagy) are actively involved in the cellular management of α-synuclein. Not surprisingly, the respective inhibition of chaperone, proteasome, or autophagy function enhances the accumulation of α-synuclein and simultaneous inhibition of these systems promotes a synergic formation of α-synuclein-positive inclusions (Rott et al., 2008). In turn, the accumulation of α-synuclein can exert reciprocal effects on the various QC systems. For example, components of the Hsp70 chaperone system (Hsp70 and Hsp40) can be depleted through their sequestration within α-synuclein-positive aggregates (Auluck et al., 2002). Further, aggregated α-synuclein species can selectively interact with the components of the proteasome complex and concomitantly inhibit its function (Snyder et al., 2003). Finally, α-synuclein can also inhibit macroautophagy (otherwise referred to as “autophagy”). This was exemplified in a mouse model of PD where α-synuclein is over-expressed. These mice exhibit signs of autophagy inhibition that apparently occurs at a very early stage of autophagosome formation (Winslow et al., 2010). Supporting this, another group recently reported that α-synuclein over-expression can result in disrupted localization and mobilization of Atg9, a multi-spanning membrane protein whose associated vesicles are important sources of membranes for the synthesis of early autophagosomes, thereby providing a mechanism for α-synuclein-induced inhibition of autophagy (Yamamoto et al., 2012). Besides macroautophagy, α-synuclein can also affect the function of chaperone-mediated autophagy (CMA), a specialized form of lysosomal degradation where proteins like α-synuclein containing a particular penta-peptide motif related to KFERQ are transported across the lysosomal membrane via the action of the integral membrane protein LAMP-2A that is assisted by both cytosolic and lumenal hsc70 (Klionsky et al., 2011) (Figure 1). Membrane bound α-synuclein species harboring disease-associated mutations or those modified by DA bind to the CMA lysosomal receptor with high affinity but are poorly translocated, which allow them time to seed the formation of oligomeric complexes on the membrane surface that consequently places the translocation complex under siege (Cuervo et al., 2004; Martinez-Vicente et al., 2008). The resulting blockage of uptake and degradation of CMA substrates further amplifies the burden of misfolded protein load (including α-synuclein) for the cell and perpetuates a vicious cycle of protein aggregation that can lead to the demise of neurons, especially dopaminergic neurons. Consistent with this, CMA inhibition following L-DOPA treatment is more pronounced in ventral midbrain cultures containing dopaminergic neurons than in non-DA producing cortical neurons (Martinez-Vicente et al., 2008).

It is apparent from the above that the chaperone, ubiquitin-proteasome, and autophagy pathways all have a role to play in the biogenesis of α-synuclein-positive LBs and thereby PD. Accordingly, pharmacological or genetic inhibition of these protein QC pathways (particularly those involved in protein degradation) in animal models should in theory be able to recapitulate the disease process. Although controversial, several groups have indeed reported evidence of SN dopaminergic neurodegeneration and associated locomotion deficits as well as the presence of neuronal inclusions in rodents subjected to subcutaneous injections of either naturally occurring or synthetic proteasome inhibitor (Lim, 2007). Using a genetic approach, Bedford et al. (2008) made similar observations in mice that are selectively depleted of functional proteasomes in their SN, which exhibit extensive nigro-striatal degeneration that is accompanied by the presence of α-synuclein-positive LB-like inclusions. Likewise, targeted genetic ablation of essential autophagy components (i.e., Atg5 or Atg7) in neural cells of mice also results in extensive neurodegeneration and widespread inclusion pathology (Hara et al., 2006; Komatsu et al., 2006). Moreover, when autophagy is selectively disrupted in midbrain dopaminergic neurons, it results in abnormal presynaptic accumulation of α-synuclein that is accompanied by dendritic and axonal dystrophy, reduced striatal DA content, and the formation of somatic and dendritic ubiquitinated inclusions (Friedman et al., 2012). Importantly, these conditionally knockout mice exhibit significant age-dependent loss of nigral dopaminergic neurons that is accompanied by markedly decreased spontaneous motor activity and coordination relative to controls (Ahmed et al., 2012; Friedman et al., 2012). Together, these studies strongly support a role for proteasomal and lysosomal dysfunction in disease pathogenesis.

Perhaps the most direct evidence to date linking lysosomal impairments to PD is the demonstration that loss-of-function mutations in a gene encoding for the lysosomal P-type ATPase named *ATP13A2* cause a juvenile and early-onset form of parkinsonism (albeit one that is also characterized by pyramidal degeneration and dementia) (Ramirez et al., 2006). What is particularly noteworthy is that the expression and toxicity of α-synuclein is enhanced in patient-derived fibroblasts as well as in ATP13A2-silenced primary mouse neurons as a result of impaired lysosomal degradation capacity arising from deficient ATPase function (Usenovic et al., 2012). Importantly, silencing of endogenous α-synuclein ameliorated the toxicity in neurons depleted of ATP13A2, suggesting that ATP13A2-induced parkinsonism may be contributed by α-synuclein accumulation as a result of functional impairments of the lysosome. Supporting this, over-expression of wild type ATP13A2 suppresses α-synuclein-mediated toxicity in *C. elegans* while knockdown of ATP13A2 expression promotes the accumulation of misfolded α-synuclein in the animal (Rappley et al., 2009). Collectively, these studies further emphasize a patho-physiological link between lysosomal dysfunction and α-synuclein in dopaminergic neurodegeneration.

Besides α*-synuclein* and *ATP13A2*, several other PD-linked genes have also been associated directly or indirectly to either the ubiquitin-proteasome and/or autophagy-lysosome systems. Among these is parkin, a ubiquitin ligase (E3) that several groups including ours have shown to be involved in both the proteasomal and autophagy QC systems. Parkin is a unique multifunctional E3 member capable of mediating multiple forms of ubiquitin modifications including mono-ubiquitination, K48-linked (proteasome-associated) and K63-linked (proteasome-independent) polyubiquitination (Dawson and Dawson, 2010). The fate of a parkin substrate thus depends on the ubiquitin topology it receives. For example, while parkin-mediated (presumably K48-linked) ubiquitination of the substrates AIMP2 and PARIS coupled them to proteasome-mediated degradation (Ko et al., 2005; Shin et al., 2011), we and others have shown that parkin-mediated K63-linked ubiquitination of synphilin and mutant DJ-1 promotes their aggregation into inclusion bodies and their subsequent removal via autophagy (Lim et al., 2005; Olzmann et al., 2007). Thus, parkin-mediated protein QC appears to involve both the proteasome and lysosome degradation machineries. Accordingly, one could envision that disease-associated parkin mutations that result in the functional disruption of its activity can lead to the toxic accumulation of both soluble (that would otherwise be cleared by the proteasome) and/or aggregated forms (that would otherwise be cleared by autophagy) of its broad spectrum of substrates. Not surprisingly, the functional assignment of parkin as a ubiquitin ligase at the turn of the century had fueled intense research into the role of the ubiquitin-proteasome system (UPS) in PD pathogenesis, which arguably has become less “trendy” now. In recent years, the attention of parkin-UPS axis has shifted toward its ability to remove damaged mitochondria via a specialized form of autophagy known as “mitophagy” (Narendra et al., 2008), a term originally coined by Lemasters (2005). Accordingly, impairment in mitochondrial QC due to failed mitophagy in parkin-deficient neurons is now thought to be a key mechanism that predisposes them to degeneration. Understanding precisely how parkin regulates mitochondrial QC and how disruptions in this process contribute to PD pathogenesis is a current “hot” topic amongst PD researchers that has helped rekindle widespread interest in an “old” pathogenic culprit.

## Mitochondrial Dysfunction and PD

A role for mitochondria dysfunction in the pathogenesis of PD has long been appreciated. The idea that mitochondrial dysfunction could contribute to the development of PD originates from the observation by Langston et al. (1983) in the early eighties that drug abusers exposed to 1-methyl-4-phenyl-1,2,3,4-tertahydropyridine (MPTP), an inhibitor of mitochondrial complex I function, display motoric features that bear uncanny resemblance to those exhibited by sporadic PD patients. Further, through post-mortem analysis performed as early as 1989, several groups have recorded a significant reduction in the activity of mitochondrial complex I as well as ubiquinone (co-enzyme Q10) in the SN of PD brains (Schapira et al., 1989; Shults et al., 1997; Keeney et al., 2006). Consistent with the proposed role of mitochondrial dysfunction as a pathogenic driver of PD, mitochondrial poisoning through the administration of toxins such as MPTP and rotenone recapitulates PD-related features in animals and represents a popular strategy to model the disease (Dauer and Przedborski, 2003). Interesting, whereas dopaminergic neurodegeneration induced by MPTP can be explained by the fact its conversion into its toxic principle MPP+ endow it with the selectivity for dopaminergic neurons (by virtue of the exquisite affinity MPP+ has for DA transporters), rotenone by comparison is more broadly distributed in the brain following its administration into animals (Betarbet et al., 2000). Despite the more systemic distribution of rotenone in treated animals, its toxicity is mostly confined to dopaminergic neurons, suggesting that dopaminergic neurons are uniquely susceptible to complex I inhibition (Betarbet et al., 2000). Similarly, impairment of mitochondrial homeostasis via genetic ablation of TFAM, a mitochondrial transcription factor that plays a critical role in maintaining mitochondrial DNA, in dopaminergic neurons of mice results in an energy crisis and neurodegeneration (Sterky et al., 2011). Moreover, the neuronal loss is progressive and accompanied by intraneuronal cytoplasmic inclusions (albeit not α-synuclein-positive). This interesting mouse model that rather faithfully recapitulates the salient features of PD is popularly known as the “MitoPark” mouse, although critics maintain that this model is of limited therapeutic utility as the mutation is not based on human PD genetics. Notwithstanding this, these above studies when taken together provide compelling support for a role of mitochondrial dysfunction in PD pathogenesis.

**Figure 2:**
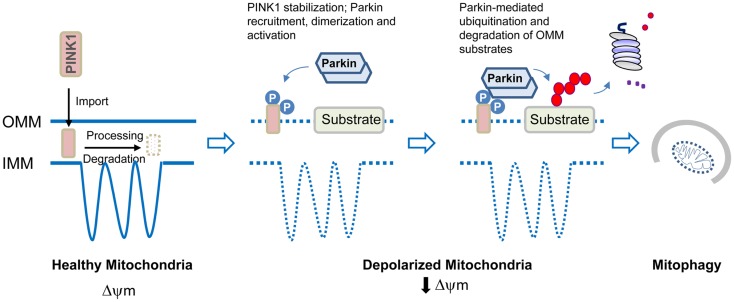
**Model of parkin/PINK1-mediated mitophagy**. In healthy mitochondria, PINK1 imported through the outer mitochondrial membrane (OMM) is rapidly processed and degraded. Upon mitochondrial depolarization, PINK1 stabilization on the OMM leads to its dualautophosphorylation on Ser228 and Ser402. This event somehow triggers parkin recruitment, self-association, and catalytic activation. Parkin ubiquitinates several proteins on the OMM that results in their degradation by the proteasome. Mitophagy induction then occurs.

Less is however known about how mitochondria become defective in PD. It is important to recognize that mitochondria are not solitary and static structures as depicted in many textbooks but rather are dynamic and mobile organelles that constantly undergo membrane re-modeling through repeated cycles of fusion and fission. In addition, the organelle also undergoes regulated turnover via mitophagy when it is damaged beyond repair. It follows that mitochondrial dysfunction can occur at different levels ranging from organelle biogenesis, fusion/fission to mitophagy. Indeed, genetic mutations that disrupt the function of mitochondrial fusion/fission regulators leads to neurodegenerative diseases such as Charcot–Marie–Tooth type 2A (Zuchner et al., 2004) and autosomal dominant optic atrophy (Alexander et al., 2000; Delettre et al., 2000) although not PD *per se*. At least for parkin-related cases, a mechanism underlying mitochondrial dysfunction has recently emerged (Figure 2). Briefly, the proposed model posits that parkin collaborates closely with another PD-linked gene known as PINK1, a mitochondrial serine/threonine kinase, to initiate the removal of depolarized/damaged mitochondria. A key initial event for mitophagy to occur is the selective accumulation of PINK1 on the outer membrane of the damaged organelle, which is otherwise prevented by a series of sequential proteolytic events in healthy mitochondria (Becker et al., 2012; Greene et al., 2012). In depolarized mitochondria, PINK1 stabilization on the outer membrane enables the protein to recruit parkin to the organelle, a process that is apparently dependent on PINK1 autophosphorylation at Ser228 and Ser402 (Okatsu et al., 2012). This event some how triggers parkin self-association (Lazarou et al., 2013), which is likely to unmask its latent activity, the consequence of which is the ubiquitination and subsequent degradation of several outer membrane protein members (Chan et al., 2011; Yoshii et al., 2011) including the pro-fusion mitofusin proteins (Poole et al., 2010; Ziviani et al., 2010). The degradation of mitofusins is probably critical to prevent unintended fusion events involving damaged mitochondria and thereby their re-entry into the undamaged mitochondrial network from occurring. Mitophagy induction then occurs, which likely involves parkin-mediated K63 ubiquitination that will help recruit the autophagy adaptors HDAC6 and p62, subsequently leading to mitochondrial clustering around the peri-nucleus region. By virtue of their association with the autophagy process, the concerted actions of p62 and HDAC6 will presumably facilitate the final removal of damaged mitochondria by the lysosome (Ding et al., 2010; Geisler et al., 2010; Lee et al., 2010). Interestingly, according to a recent report from Mizushima’s lab, mitophagosomes may be generated in a *de novo* fashion on damaged mitochondria to initiate their removal. The authors demonstrated that parkin recruitment on the mitochondria induces the formation of ULK1 (Atg1) puncta (an upstream nucleation step of the hierarchical autophagy cascade) and Atg9 structures (Itakura et al., 2012), although it remains unclear mechanistically how parkin participates in the *de novo* synthesis of isolation membrane.

Although several groups have demonstrated that PD-associated parkin mutants are defective in supporting mitophagy due to distinct problems at recognition, transportation, or ubiquitination of impaired mitochondria (Lee et al., 2010; Matsuda et al., 2010), a pertinent question to ask is whether deficient mitochondrial QC is relevant at all to the large number sporadic PD cases where parkin is not mutated. Although this remains to be established, it is noteworthy to mention that we and others have previously shown that parkin dysfunction could arise in the PD brain in the absence of apparent mutations. This could be a result of stress-induced biochemical alterations including oxidation and nitrosylation, post-translational modifications or aberrant protein–protein interaction that can either alter the catalytic function of the E3 ligase directly, or indirectly through promoting its aggregation or degradation (LaVoie et al., 2005; Wang et al., 2005). Interestingly, normal parkin in the brain also becomes progressively more detergent-insoluble (and therefore non-functional) with aging (Pawlyk et al., 2003), which may provide an explanation to why age represents a risk factor for PD. In all these cases, the loss of parkin function is expected to compromise the efficiency of parkin-mediated mitophagy, amongst other parkin-regulated events. Thus deficient mitochondrial QC may not necessarily be restricted to cases where parkin (or PINK1) is overtly mutated. Moreover, it is also becoming increasingly clear that various other PD-linked proteins that may appear to have disparate functions could all influence mitochondrial homeostasis, directly or indirectly (Lim et al., 2012). For example, several groups including ours have recently found that the disease-associated LRRK2 G2019S mutant can trigger marked mitochondrial abnormalities when over-expressed in cultured cells and *in vivo* (Ng et al., 2012; Niu et al., 2012; Wang et al., 2012). Corroborating these findings, Mortiboys et al. (2010) demonstrated that cells derived from LRRK2 G2019S patient exhibited similar mitochondrial abnormalities, a phenotype that was also shared by neural cells derived from LRRK2 patients via induced pluripotent stem cell technology (Cooper et al., 2012). More recently, investigators from Chu Lab reported that LRRK2 elicited calcium imbalance and depletion of dendritic mitochondria in neurons (Cherra et al., 2013). Thus, mitochondrial dysfunction seems to be a common denominator underlying both familial and sporadic forms of PD.

## Crosstalk between the Protein and Mitochondrial QC Systems

What is clear from the above description is that the crosstalk between mitochondrial QC sensor and the autophagy apparatus needs to be tightly regulated to ensure that the pool of organelles available to energy-demanding cells such as neurons are bio-energetically competent. Failure in autophagy will therefore be expected to affect mitochondrial QC as much as it will affect protein QC. Given this, it is perhaps not surprising to note the frequent co-occurrence of mitochondrial abnormalities and inclusion bodies, the latter appearance is arguably an indication of failed autophagy function. At the same time, mitochondrial QC is also intimately intertwined with UPS machinery. After all, the initial stages of mitophagy involves the ubiquitination and consequent degradation of mitofusin of several mitochondrial outer membrane proteins such as Tom 20, Tom 40, Tom 70, and Omp 25 (Chan et al., 2011; Yoshii et al., 2011). To facilitate this en-bloc removal of mitochondrial proteins, parkin activates the UPS upon translocation to the mitochondria. This occurs by means of the enrichment of the proteasome-linked K48-linked ubiquitination of targeted proteins and recruitment of the proteasome to the mitochondria, the process of which is thought to lead to the rupturing of the mitochondrial outer membrane in preparation for mitophagy induction (Chan et al., 2011; Yoshii et al., 2011). Besides mitophagy regulation, a recent study revealed that parkin can also potentially regulate mitochondrial biogenesis by regulating PGC-1α expression indirectly through its ability to down-regulate PARIS (Shin et al., 2011), which otherwise represses PGC-1α expression transcriptionally (Scarpulla, 2008). Again, proteasome function is at play here as the degradation of PARIS occurs via the proteasome machinery following its ubiquitination by parkin (Shin et al., 2011).

Besides parkin, other UPS-associated enzymes also participate in mitochondrial homeostasis. For example, the E3 members, MARCH-V and MULAN, as well as a deubiquitinating enzyme, USP30 are active regulators of mitochondrial dynamics (Livnat-Levanon and Glickman, 2011). All of these UPS-associated members reside on the mitochondrial outer membrane and collectively, they provide a link between the organelle and the proteasome. Interestingly, inclusion bodies formed in mice depleted of functional proteasomes contain mitochondria, suggesting a potential (albeit intriguing) role for the organelle in the biogenesis of protein inclusions (Bedford et al., 2008). In a reciprocal fashion, mitochondrial dysfunction can also impact proteasome function. Notably, energy in the form of ATP is required to assemble the proteasome complex as well as to drive the UPS machinery. Conceivably, in times of energy crisis, the UPS may not function optimally, which in turn may affect mitochondrial QC. Furthermore, an inevitable consequence of aerobic respiration is the generation of reactive oxygen species (ROS), which can modify components of the UPS itself, including E3 ligases that utilize active thiol groups. As alluded earlier, parkin is particularly susceptible to oxidative modification, which alters its solubility and promotes its aggregation in a manner analogous to that brought about by several of its missense mutations (LaVoie et al., 2005; Wang et al., 2005). Similarly, the 19S regulatory cap of the proteasome also appears to be especially sensitive to oxidation. Indeed, the S6/Rpt3 subunit of the 19S cap has been identified to be a major target of carbonylation in cells exposed to inducers of ROS (Ishii et al., 2005). That UPS components are prone to oxidative modification is somewhat paradoxical, as they are ones in the first place responsible for the efficient clearance of proteins damaged by oxidation. Interestingly, a very recent report demonstrated that chronic mitochondrial impairment results in the disassembly of 26S proteasome via calpain-mediated cleavage of Rpn10 that is accompanied by a concomitant increase in 20S proteasome level and activity (Huang et al., 2013). The authors posit that the increased function of 20S proteasomes, which can degrade proteins in an unregulated and energy-independent manner, may help the cell clear randomly unfolded oxidized proteins that would otherwise build up as a result of mitochondrial dysfunction. Obviously, this strategy is beneficial only for the short-term, i.e., if chronic, unregulated protein degradation will be detrimental to cellular survival. Taken together, it is apparent that the UPS, autophagy, and mitochondrial systems interact with, and exert reciprocal effects on one another, and that ROS generated by mitochondrial respiration can modify the function of these systems thereby adding another layer of complexity to an already complex relationship.

## Oxidative Stress and PD

The production of ROS is intimately associated with mitochondrial function as well as with its dysfunction. As mentioned earlier, ROS generation represents an inevitable consequence of mitochondrial respiration. During the process of aerobic respiration, partial reduction of molecular oxygen to superoxide anion $\left({\text{O}}_{2}^{-}\right)$ occurs when electrons leak from the electron transport chain or ETC (particularly at complex I). This free radical can be converted to the highly reactive hydroxyl radical (OH^•^) via an iron-catalyzed reaction known as Fenton reaction, or to peroxynitrite (ONOO^−^) upon reaction with nitric oxide (NO). Both hydroxyl radical and peroxynitrite are potent oxidants that can cause marked cellular damage by reacting with proteins, lipids, and nucleic acids. Further, these reactive species may also target the ETC, which results in a feed forward cycle of increasing oxidative stress and injury.

The brain is often thought to be particularly susceptible to oxidation-induced damage because of its high metabolic rate and its relatively reduced capacity to replenish its post-mitotic neuronal populations compared with other organs. For SN dopaminergic neurons, the vulnerability toward oxidative stress is further enhanced by the abundance of redox-active iron in this region of the brain, as well as by the presence of DA, whose oxidation products are potentially cytotoxic (Graham, 1978). Notably, several groups have reported that markers for lipid peroxidation (including 4-hydroxynonenal and malondialdehyde), protein carbonyl modifications and even DNA and RNA oxidation are markedly elevated in the SN of post-mortem PD brains (Alam et al., 1997a,b; Zhang et al., 1999), and that these ROS-induced events are accompanied by a dramatic depletion of reduced glutathione (presumably leading to a considerably weakened antioxidant defense system) (Sian et al., 1994). As mentioned earlier, mitochondrial poisons that recapitulates PD features in humans and animals alike often target complex I, the impairment of which enhances superoxide production and thereby the formation of highly reactive free radicals that can initiate neuronal death. Importantly, oxidative damage and nigral dopaminergic neurodegeneration appears to correlate in a temporal manner in these models, suggesting a causal role of oxidation-induced stress in PD pathogenesis (McCormack et al., 2002; Peng et al., 2005).

Although the Redox chemistry of DA and the abundance of iron in SN dopaminergic neurons may underlie their heightened level of oxidative stress compared to other neuronal subtypes, another tantalizing culprit may be a unique channel type that resides on nigral dopaminergic neurons known as L-type Ca^2+^ channels. Unlike their counterparts in the Ventral Tegmental Area (VTA), SN dopaminergic neurons use L-type Ca^2+^ channels to help maintain autonomous pace-making (Chan et al., 2007). Because L-type Ca^2+^ channels are open most of the time (as they are open at relatively hyperpolarized state), the nigral neurons would experience a significantly larger magnitude and spatial extent of Ca^2+^ influx with time, which obviously comes with a price. Normally, the level of intracellular Ca^2+^ is under very tight homeostatic control by the actions of ATP-dependent pumps whose operations are metabolically expensive. A sustained entry of Ca^2+^ intonigral neurons would presumably work the mitochondria machinery harder and concomitantly raise the level of ROS that would predispose them to oxidative stress-induced degeneration. Indeed, in mice engineered to carry a mitochondrial-localized redox-sensitive form of GFP, the basal oxidation as measured by this reporter is significantly higher in SN dopaminergic neurons relative to their VTA counterparts. Importantly, the enhancement of which can be lowered simply by the administration of L-type Ca^2+^ channel antagonists into these transgenic mice (Guzman et al., 2010). The “L-type Ca^2+^ hypothesis” is certainly an attractive proposition to explain the unique vulnerability of SN dopaminergic neurons toward degeneration. Moreover, neurons in the locus ceruleus region that are also lost in the PD brain are similarly autonomous pacemaker dependent on the activity of L-type Ca^2+^ channels (Williams et al., 1984). However, the hypothesis is not an adequate explanation for all the susceptible sites in the PD brain, which extend beyond the dopaminergic and noradrenergic systems (Braak et al., 2003).

If the autophagy and UPS models of PD are supported by disease-linked ATP13A2 and parkin mutations respectively, then mutations in the redox-sensitive protein, DJ-1, which causes an early-onset form of PD, would provide the genetic support for the role of oxidative stress in PD pathogenesis. DJ-1 is thought to operate as an atypical peroxiredoxin-like peroxidase that is capable of scavenging mitochondrial H_2_O_2_ (Canet-Aviles et al., 2004). Consistent with this, increased levels of H_2_O_2_ in mitochondria can be isolated from DJ-1 knockout mice (Andres-Mateos et al., 2007). Notably, a pool of DJ-1 is known to be localized to the mitochondria (Canet-Aviles et al., 2004; Zhang et al., 2005), suggesting a functional link between DJ-1 and the organelle. Moreover, loss of DJ-1 function promotes mitochondrial fragmentation in a variety of cells including lymphoblast cells derived from DJ-1 patients and sensitizes them toward oxidative stress-induced death (Irrcher et al., 2010; Krebiehl et al., 2010; Thomas et al., 2011), a phenotype that can be rescued by restoration of functional DJ-1 expression or by scavengers of ROS (Irrcher et al., 2010; Thomas et al., 2011). Interestingly, a recent study suggest that DJ-1 enhances ERK-dependent mitophagy in the presence of the parkinsonian neurotoxin rotenone and in so doing protects dopaminergic neurons against toxin-induced apoptosis (Gao et al., 2012). Accordingly, the absence of DJ-1 may predispose dopaminergic neurons to mitochondrial dysfunction and oxidative stress-induced degeneration. Indeed, DJ-1-deficient animals are hypersensitive to pharmacological inducers of oxidative stress (Kim et al., 2005; Menzies et al., 2005; Meulener et al., 2005; Park et al., 2005; Yang et al., 2005; Manning-Bog et al., 2007). Consistent with this, dopaminergic neurons derived from *in vitro* differentiated DJ-1-deficient embryonic stem cells display decreased survival and increased sensitivity to oxidative stress (Martinat et al., 2004). Importantly, the ablation of DJ-1 expression results in the amplification of basal oxidant stress in SN dopaminergic neurons (Guzman et al., 2010).

Finally, it is important to recognize that besides intrinsic sources of ROS, oxidative radicals can also come extracellularly from activated glial cells, which is well documented in affected regions of the PD brain, as well as in genetic and toxin-induced models of PD (Hald and Lotharius, 2005). Although glia-mediated inflammatory events are often perceived as secondary to intrinsic events happening in susceptible neurons, they can aggravate and/or perpetuate the pathogenic outcomes and as such may play an instrumental role in promoting neuronal cell death. Indeed, the role of neuro-inflammation is regaining its prominence in the field as more and more researchers are now focusing on non-cell autonomous forms of death in neurodegenerative diseases (for a recent review, please refer to Hirsch et al., 2012).

## Concluding Remarks – Making Sense Out of the Apparent Chaos?

**Figure 3:**
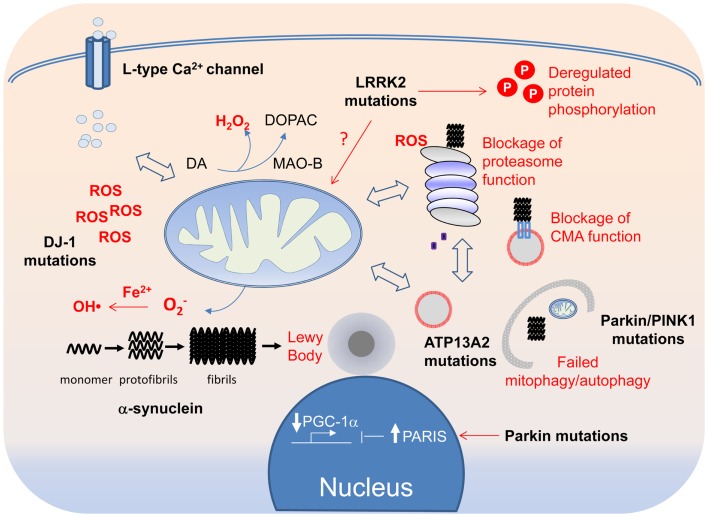
**A tapestry of molecular events in PD pathogenesis**. Disruption of mitochondrial and protein QCs can arise from overt PD-linked genetic mutations or through oxidative modifications of their components by ROS, the levels of which can be elevated by DJ-1 mutations, Fe^2+^-mediated Fenton reaction or increased Ca^2+^ influx through the L-type Ca^2+^ channel. Because of the crosstalk that exists between the QC systems, each can in turn affect the other in a reciprocal fashion. Aberrant mitochondrial and protein QCs and redox imbalance all can promote the formation of α-synuclein protofibrils and fibrils, which in turn can block the function of the proteasome and CMA. Aggregated α-synuclein species, if not cleared in a timely fashion, can also seed the formation of LBs, which can be physically obstructive to neuronal function if allowed to grow.

Most readers would agree after reading the description above that the molecular events underlying PD pathogenesis is really complex (Figure 3). Indeed, even the genes associated with PD, which otherwise give the disease a tractable etiology, are so disparate in function that at first sight, they seem to have little things (if at all) in common. This is quite unlike the situation in Huntington’s disease, which can be traced to a single genetic defect (i.e., mutations in the *Huntingtin* gene) or familial Alzheimer’s disease where the majority of the disease-linked genes are clustered around the amyloid precursor protein processing pathway. Although disruptions in protein and mitochondrial QC are consistently implicated in PD pathogenesis and are generally accepted to be the key pathogenic drivers, additional pathogenic events that have recently emerged (or are emerging) include aberrant protein phosphorylation, endosome recycling, and lipid metabolism look set to complicate the picture. Furthermore, as we have discussed, the implicated pathways often act in a reciprocal fashion to influence one another. Moreover, it is also becoming increasingly clear that each of the PD-linked gene products, when dysfunctional, can exert effects on multiple pathways either directly or indirectly. Thus, no matter how upbeat one can be for a favored PD-related pathway, it is highly unlikely to be the only pathway involved in disease pathogenesis. To use an analogy regarding our current knowledge about the molecular events underlying PD pathogenesis – it seems like we are looking at a tapestry but on its reverse side where all the different colored threads are interwoven in a seemingly chaotic fashion. Undeniably, it is a significant challenge to make sense out of the apparent chaos. Nonetheless, we certainly have a better grasp of the pathogenic events happening in the PD brain these days than we have before as a result of concerted efforts in the past decade or so by many investigators around the world in unraveling the molecular causes of the disease. Although we remain uncertain about the initiating event, it is worthy to note that the myriad of pathways proposed to be involved in disease pathogenesis appears to be converging rather than diverging from each other. It is therefore perhaps not surprising to see that various PD-linked genes with apparently different function can directly or indirectly affect the same event (e.g., mitochondrial dysfunction). Paradoxically, recognizing that PD pathogenesis is a complex process may be the first step toward understanding how the tapestry of pathogenic events is weaved together.

## Conflict of Interest Statement

The authors declare that the research was conducted in the absence of any commercial or financial relationships that could be construed as a potential conflict of interest.
